# Development of Actuators for Repairing Cracks by Coating W Wires with Reactive Multilayers

**DOI:** 10.3390/ma15030869

**Published:** 2022-01-24

**Authors:** Gabriel Santos Silva, Lukasz Maj, Jerzy Morgiel, Maria Teresa Vieira, Ana Sofia Ramos

**Affiliations:** 1 University of Coimbra, CEMMPRE, Department of Mechanical Engineering, Polo II, R. Luís Reis Santos, 3030-788 Coimbra, Portugal; gabriel.silva.95@outlook.com (G.S.S.); teresa.vieira@dem.uc.pt (M.T.V.); 2 Poland Institute of Metallurgy and Materials Science, Polish Academy of Sciences, Reymonta 25, 30-059 Krakow, Poland; l.maj@imim.pl (L.M.); j.morgiel@imim.pl (J.M.)

**Keywords:** multilayer thin films, sputtering, TEM (transmission electron microscopy), self-propagating reactions, liquid phase actuators

## Abstract

The aim of this research work was to optimize the coating of tungsten wires with reactive multilayer thin films and promote an exothermic self-propagating reaction. The ultimate goal is to use this heat to liquify low melting temperature materials, and thus block crack propagation in metallic materials. Ni/Me (Me = Al, Ti) multilayers were deposited by a DC (direct current) magnetron sputtering onto tungsten wires with diameters of 0.05 and 0.20 mm. The depositions were carried out to obtain films with near equiatomic average chemical composition and a modulation period (bilayer thickness) between 20 and 50 nm. The cross-section of the films was analyzed using electron microscopy before and after electrical ignition. A new substrate holder was developed to improve the quality of the Al/Ni films, allowing a reduction in the defects previously observed. The Ni/Ti thin films showed no discernible defects, regardless of the substrate holder. However, after ignition, the Ni + Ti reaction occurred in a non-self-propagating mode. Passing an electric current through a wire (ϕ = 0.05 mm) coated with an Al/Ni thin film, promoted a flash of light that was associated with the start of a self-propagating reaction. The reaction product was a B2-AlNi intermetallic phase. W wires coated with reactive multilayers may contribute to crack filling, and have potential to be self-healing actuators.

## 1. Introduction

Me1/Me2 (Me = metal) reactive multilayers are a class of energetic materials that have been widely studied in recent decades [1,2,3]. In particular, Al/Ni multilayers with a nanometric period (bilayer thickness or spacing) have drawn a high level of interest among the scientific community [4,5,6]. The Al + Ni → AlNi heat of reaction is −59 kJmol^−1^, and temperatures above 1600 °C can be attained [2]. Other Me1–Me2 reactive systems, such as Ni–Ti, have also been studied [7]. NiTi shape memory alloy thin films with promising mechanical properties can be prepared from Ni/Ti multilayers [8,9]. Under certain conditions, the exothermic reaction between Me1 and Me2 may proceed along a self-propagating high temperature synthesis (SHS) path [2]. The majority of reactive multilayers have been deposited by physical vapor deposition, namely by sputtering and electron-beam evaporation, with most work in the 21st century relying on DC magnetron sputtering [6].

The exothermic and self-propagating characteristics of reactive multilayers makes them promising as highly localized heat sources for several applications [2,6]. Different approaches regarding the use of reactive multilayers, in the form of thin films or foils, to promote or enhance joining have been reported in the literature. For several years, free-standing Al/Ni foils have been used as heat sources capable of melting solder and brazing alloys and, thus, promote joining–reactive brazing, e.g., [10,11,12]. A different approach consists of taking advantage of the nanometric character, and the heat released by reactive multilayer thin films, to enhance the diffusing bonding process–reaction assisted diffusion bonding, e.g., [13,14]. In this case, the reactive multilayers are directly deposited onto the materials being joined. It should be noted that during the self-propagation of thick free-standing foils, thermal conduction along the foil is the dominant mode of heat loss [2]. However, for multilayer thin films, heat transfer into the substrate via conduction or into the surrounding atmosphere via conduction, convection, and/or radiation must be considered [2]. In fact, heat dissipation into the substrate can hinder SHS. According to Rabinovich et al. [15], for Al/Ni films on a substrate, SHS could only occur in films with a sufficiently large number of alternated layers.

Although reactive multilayers have been mostly used, so far, as localized heat sources to promote and enhance joining, they could also have the potential for self-healing applications. In the last application, the heat released would promote the melting of a repairing material. In this context, a wire coated with a reactive multilayer film and a repairing material with a low melting temperature can constitute an actuator of a self-healing system. The ignition of the reactive multilayer, through the application of an electrical discharge, should give rise to enough heat to improve the flowability of the selected repairing material, essential to fill any material failures and stop crack propagation.

The coating of wires with reactive multilayer thin films with nanometric period is a challenging task, because the deposition of alternating nanolayers is carried out onto the curved surface of wires with diameters below 0.5 mm [16]. The challenge is even greater in the case of multilayer thin films from the Al–Ni system, because aluminum and nickel react at quite low temperatures, e.g., [3,17]. In a recent paper, Al/Ni multilayers were deposited onto different substrates; the authors concluded that the surface topography, interface roughness, and the phase transformation of the multilayer films show strong dependence on the substrates [18]. The period is also important as it can determine the occurrence of a self-propagating reaction, as well as the properties of nanolayered films. The velocity of self-propagating reactions in free-standing foils increases with the decrease in the period up to a maximum value, after which it decreases as the period further decreases [2]. In the case of Al/Ni multilayers, the reactivity peak occurs for a period close to 25 nm [2]. For higher periods, the diffusion distances increase, thus the reaction velocity decreases. The maximum self-propagating bilayer thickness, defined as the quench limit, was studied in detail by Kittell et al. [19] for Al/Pt multilayers on different substrates, and also as free-standing foils. On the other hand, as the period decreases, the effect of intermixing becomes more pronounced and the reaction velocity can decrease for short periods; in certain cases, it even can impede the reaction to become self-sustained. As the period decreases the number of interfaces increase and, due to the lower individual layer thickness, the grain size decreases, resulting in greater hardness. This behavior is observed in several metallic multilayer systems, namely in Ag/Ni and Al/Ni multilayers with nanometric periods [20,21].

The objective of the present work is to optimize the coating of tungsten wires with reactive multilayer thin films, to promote an exothermic self-propagating reaction between Ni and Me (Me = Al, Ti). These coated wires have the potential to be used, together with a low melting point material, in the development of liquid phase actuators responsible for repairing cracks. This work is part of an ambitious project whose objective is to contribute to the possibility of self-healing in metallic materials using a twofold approach: (i) a crack sensor [22], and (ii) an actuator.

## 2. Materials and Methods

### 2.1. Deposition Technique

**Figure 1:**
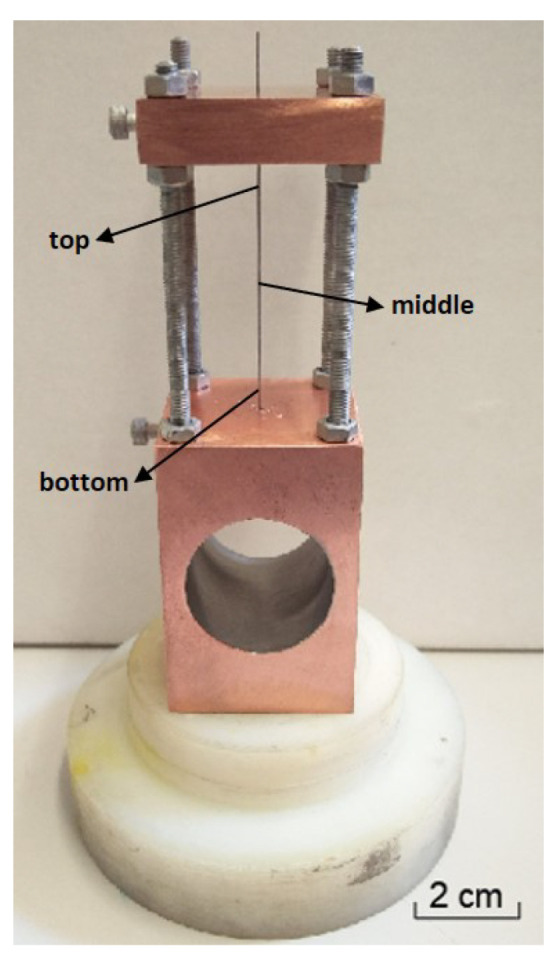
The new substrate holder dedicated to the coating of wires.

Ni/Me (Me = Al, Ti) multilayer thin films were deposited onto tungsten wires (ϕ = 0.05 and 0.20 mm) and Si substrates via DC magnetron sputtering from two targets (Ni and Me). The Ni target contained a few percent of vanadium making it non-magnetic, and enabling more stable depositions. A stainless steel sheet shield was placed between the two sputtering targets to minimize cross-contamination and avoid the mixing of atomic fluxes. The power in each target was adjusted to obtain a near equiatomic average chemical composition. Me was selected as the bottom layer to improve adhesion to the substrate, and the top layer was always Ni to reduce surface oxidation. The average chemical composition was evaluated using energy dispersive spectroscopy (EDS, Oxford Instrument, Oxfordshire, UK) in multilayer thin films with short period (Λ, bilayer thickness), previously deposited onto Si substrates (Λ < 10 nm). Once the desired chemical composition was achieved, Ni/Me multilayer thin films were deposited onto W wires with different diameters using the same deposition parameters, apart from the rotation speed of the substrates. Multilayer thin films with different periods (20 < Λ < 50 nm) were produced by varying the substrate holder rotation speed. The multilayer periods were selected based upon previous reaction-assisted diffusion bonding experiments that used Ni/Me multilayers deposited using the same sputtering equipment [3,14]. To obtain the desired modulation periods, the substrate holder rotation speed varied between 1.9 and 3.5 rpm. After attaining a base pressure below 5 × 10^−4^ Pa, argon was introduced in the deposition chamber and the substrates were cleaned by heating, followed by ion etching. The deposition process, carried out at 0.4–0.45 Pa, started after turning on the targets’ power supplies. The deposition time varied between 30 and 32 min, and the total thickness of the multilayer thin films was close to 3.0 µm, as confirmed by profilometry using Si substrates. A new copper substrate holder was developed, which aimed at avoiding or reducing the defects observed in Al/Ni multilayer thin films. In the new substrate holder (Figure 1), both ends of the wires are in contact with copper, making heat dissipation more efficient and keeping the wires straight. In the case of the “old” substrate holder, the top end of the wire was left free (devoid of heat sink) [16].

**Figure 2:**
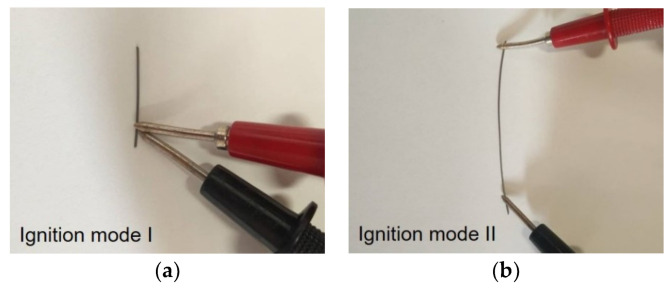
Ignition of the Ni/Me coated wires by (**a**) mode I, and (**b**) mode II.

Using the multilayer coated W wires, ignition tests were performed by applying a pulse of energy (electric source). The ignition was carried out as follows: (i) by a local electrical discharge (9 V)-mode I; and (ii) by passing a pulse of current (9 V) through the W wire-mode II (Figure 2).

### 2.2. Characterization Techniques

The microstructure of as-deposited and ignited films was studied using scanning and transmission electron microscopy (SEM and TEM), backed by chemical composition using EDS. The SEM analyses were carried out at the laboratory for scanning electron microscopy and X-ray microanalysis of the Materials Centre of the University of Porto (CEMUP), using a high-resolution microscope (FEI Quanta 400FEG ESEM/EDAX Genesis X4M (FEI Company, Hillsboro, OR, USA)). Electron imaging was performed at 15 kV accelerating voltage. Detailed TEM analyses were performed using a FEI Tecnai microscope of the Institute of Metallurgy and Materials Science, Polish Academy of Sciences (FEI Tecnai G2 FEG SuperTWIN, Eindhoven, The Netherlands), which is equipped with high angular annular dark field (HAADF) and EDAX detectors. For phase composition, selected area electron diffraction (SAED) was carried out in the TEM. Process Diffraction^®^ software (Janos Labar, Budapest, Hungary) was used for the indexation of the SAED patterns. The thin foils for TEM were prepared using a FEI Quanta 3D 200 Dual Beam Focused Ion Beam (FIB) equipped with an Omniprobe lift-out system.

## 3. Results and Discussion

### 3.1. As-Deposited

**Figure 3:**
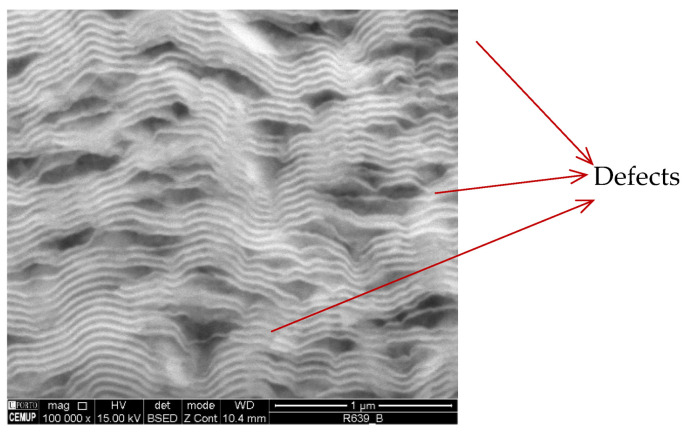
BSE SEM cross-section micrograph of an Al/Ni thin film with a period close to 50 nm deposited onto a W wire (ϕ = 0.20 mm).

In the present study, Ni/Me (Me = Al, Ti) multilayer thin films were deposited via DC magnetron sputtering onto W wires with different diameters at conditions similar to those previously selected to deposit Al/Ni thin films (Λ ≈ 30 nm) onto tungsten wires with a diameter of 0.05 mm [16]. The selection of W wires with diameters higher than 0.05 mm allows easy handling, and the defects observed in the previous work [16] might be reduced. A cross-section SEM image of an Al/Ni multilayer thin film deposited onto a W wire (ϕ = 0.20 mm) is presented in Figure 3.

**Figure 4:**
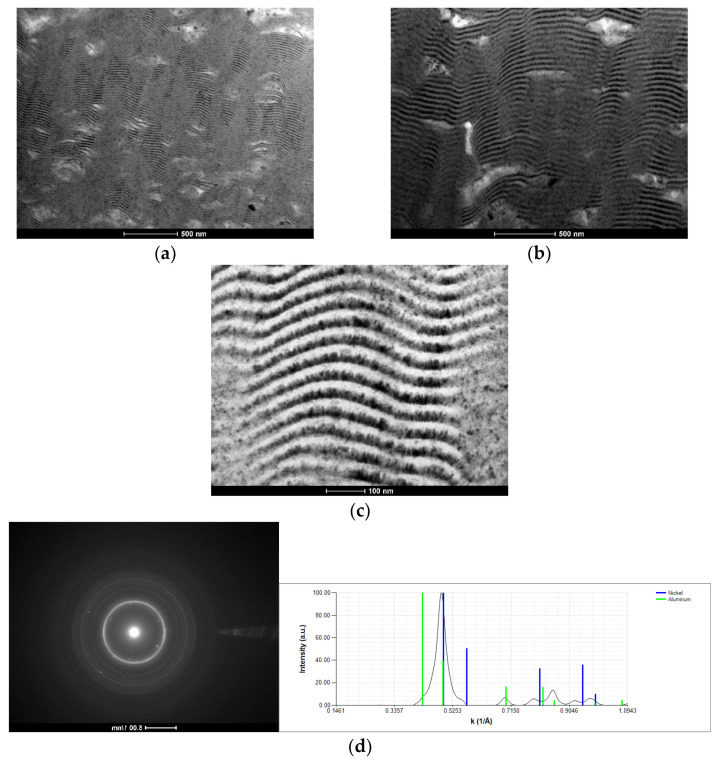

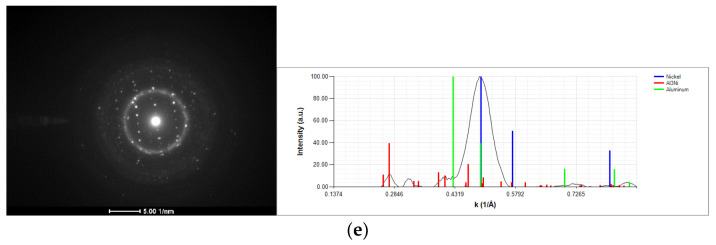
BF TEM cross-section micrographs of Al/Ni thin films with (**a**) Λ ≈ 25 nm, and (**b**,**c**) Λ ≈ 45 nm deposited onto W wires (ϕ = 0.20 mm). SAED patterns and respective indexation of the Al/Ni thin film with Λ ≈ 45 nm in zones (**d**) without, and (**e**) with defects.

The modulation period of this multilayer is close to 50 nm. The presence of several defects with submicrometric size can be identified as dark regions (rich in light elements) in the SEM backscattered electron (BSE) images. These Al-rich defects are also observed in the TEM bright field (BF) images of Al/Ni multilayer thin films with different modulation periods (Figure 4a–c). In addition to the defects, zones with well-defined alternated nanolayers can be observed together with rather undefined zones, for both periods. As the period increases the defects’ size also increases. Therefore, SAED patterns were acquired from defect-free zones, as well as from a large defect of the Al/Ni thin film with the highest period (Λ ≈ 45 nm). The SAED patterns of Figure 4d,e were indexed as Ni + Al and Ni + Al + Al_3_Ni, respectively, meaning that the defects should correspond to Al_3_Ni. The presence of the Al_3_Ni intermetallic phase can be explained by the occurrence of local reaction between Ni and excess Al, already during the deposition process without substrate cooling. It should be noted that, as referred to previously, Al and Ni react at low temperatures, particularly in multilayers with short periods [3,17]. Based on differential scanning calorimetry (DSC) measurements, for intermediate periods (~25 to ~40 nm), the reaction might occur below 200 °C [23]

**Figure 5:**
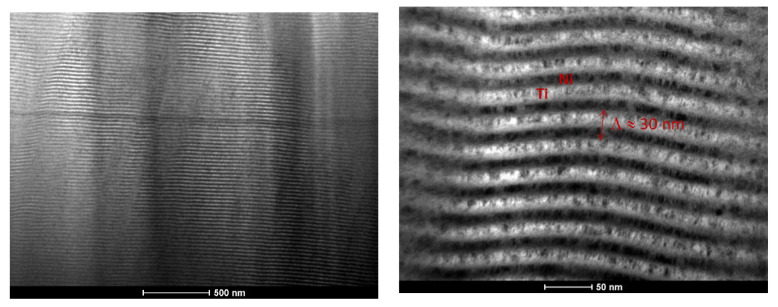
BF TEM cross-section micrograph of a Ni/Ti thin film with a period close to 30 nm, deposited onto a W wire (ϕ = 0.20 mm).

In Figure 5, a BF TEM image of a Ni/Ti multilayer thin film with Λ ≈ 25 nm deposited onto a W wire (ϕ = 0.20 mm) is shown.

The alternated Ni and Ti nanolayers can be distinguished throughout the whole thickness of the film, without such defects as those observed for the Al/Ni thin films. According to hot X-ray diffraction analyses, the reaction temperature in Ni/Ti multilayer thin films with a similar period is close to 400 °C [24]. This temperature is not attained in the substrate during the sputtering deposition process; therefore, the presence of intermetallic phases in the Ni/Ti as-deposited films was not expected. DSC experiments of Ni/Ti multilayer thin films with 30 nm bilayer thickness also indicate that the formation of intermetallic phases should not occur during the deposition process of these multilayers [25].

**Figure 6:**
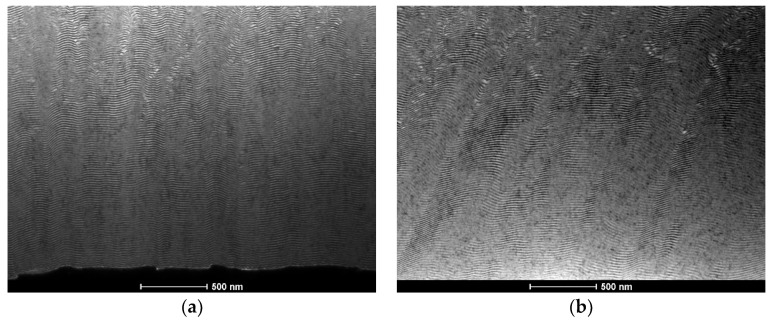
BF TEM cross-section micrographs of an Al/Ni thin film with Λ ≈ 20 nm, deposited using the new substrate holder (Figure 1). (**a**) bottom, and (**b**) top of the W wire (ϕ = 0.20 mm).

Since the Al/Ni multilayer thin films were marred by the presence of multiple defects, a new substrate holder was designed to allow for more efficient heat dissipation, i.e., also through the top of the wire, as mentioned in Section 2 (Figure 1). Using the new substrate holder, Al/Ni and Ni/Ti multilayer thin films were deposited using DC magnetron sputtering onto W wires with different diameters. BF TEM images of an Al/Ni multilayer thin film (Λ ≈ 30 nm) deposited onto a W wire (ϕ = 0.20 mm) are shown in Figure 6.

**Figure 7:**
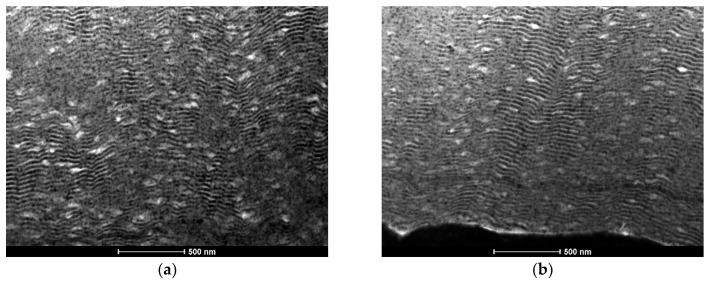
BF TEM cross-section micrographs of an Al/Ni thin film with Λ ≈ 35 nm, deposited using the new substrate holder (Figure 1). (**a**) bottom, and (**b**) top of the W wire (ϕ = 0.05 mm).

Comparing Figure 4 and Figure 6, it can be concluded that the use of the new substrate holder reduces the Al-rich defects significantly. In fact, almost defect-free films were deposited, from both the bottom and the top of the W wire (Figure 6). However, when using W wires with ϕ =0.05 mm the defects are still present in the Al/Ni films (Figure 7). During deposition, heat dissipation by conduction through the tungsten substrates is less efficient when using W wires with ϕ = 0.05 mm than with ϕ = 0.20 mm, which explains the defects observed in Figure 7. For the smallest wire diameter, the presence of defects could not be avoided, although the Al-rich defects are considerably smaller than those observed when using the “old” substrate holder. In addition, the images from the bottom and top of the wire are similar, revealing that the new substrate holder assures uniform multilayer thin films throughout the whole length of the W wires. Therefore, using the middle part of the coated wires, as presented in Figure 6 and Figure 7, ignition by mode I was attempted.

### 3.2. After Ignition

**Figure 8:**
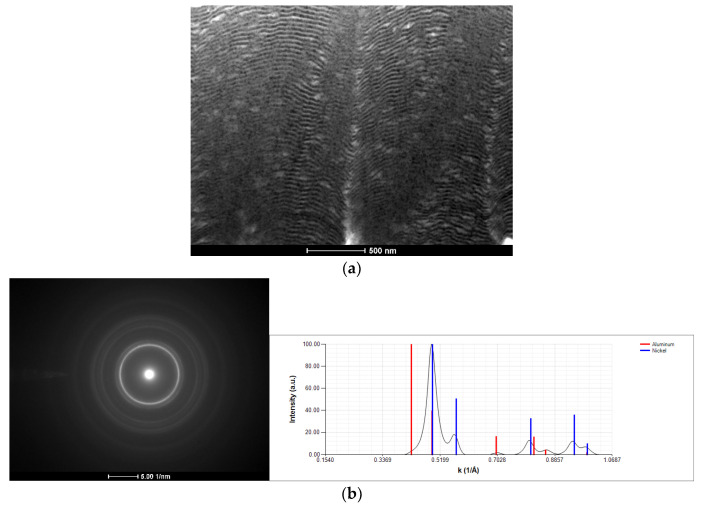
(**a**) BF TEM cross-section micrograph, and (**b**) SAED pattern and respective indexation of an Al/Ni thin film following ignition by mode I (ϕ = 0.05 mm W wire).

**Figure 9:**
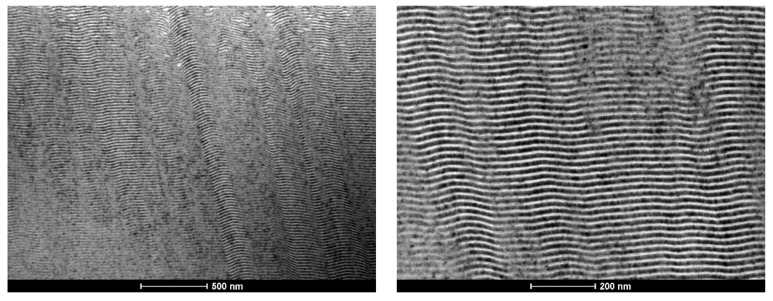
BF TEM cross-section micrographs of an Al/Ni thin film following ignition by mode I (ϕ = 0.20 mm W wire).

The local application of 9 V onto the W wire (ϕ= 0.05 mm) coated with Al/Ni multilayer thin film (Λ ≈ 35 nm) did not produce a bright flash. At the same time, the “post-reaction” microstructure has vestiges of alternated nanolayers (Figure 8), proving that self-propagation was not promoted. As expected, in the SAED pattern, non-reacted Al and Ni were identified (Figure 8b). For the Al/Ni multilayer thin film deposited onto the W wire with ϕ = 0.20 mm, which is almost defect-free, ignition by mode I was also unable to start a self-propagating reaction (cf. Figure 9). In fact, in this case a reaction did not occur at all.

Although self-propagation by mode I was previously achieved in an Al/Ni multilayer thin film deposited onto a W wire (ϕ = 0.05 mm) [16], it was not possible to reproduce it in other tests using the same conditions. The 0.05 mm diameter should be close to the limit where the heat dissipated through the tungsten substrate impedes the ability of the reaction to progress in a self-sustained way upon local ignition by mode I. Therefore, for larger diameters, it will be practically impossible to trigger a SHS reaction in Al/Ni multilayer thin films, deposited onto W wires, using this ignition mode. The use of wires with diameters larger than 0.05 mm is desirable because they are easier to handle, and because the number and size of the defects observed in the Al/Ni multilayers is significantly reduced.

**Figure 10:**
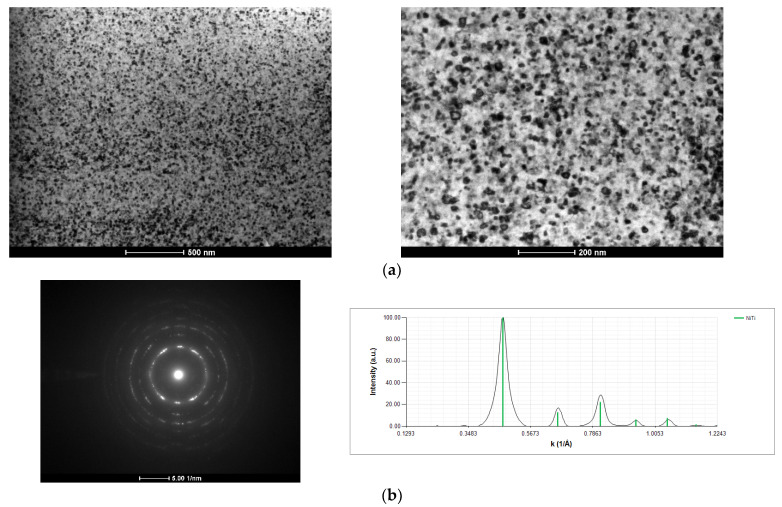
(**a**) BF TEM cross-section micrographs, and (**b**) SAED pattern and respective indexation of a Ni/Ti thin film following ignition by mode I (ϕ = 0.20 mm W wire).

The cross-section of a Ni/Ti thin film deposited onto a W wire (ϕ = 0.20 mm), after several attempts at ignition using mode I, is shown in Figure 10. Although a bright flash was never observed, the layered structure disappeared due to the local reaction and mixing of Ni and Ti. In accordance with the average chemical composition of the multilayers, the NiTi equiatomic phase was formed (Figure 10b). However, Ni and Ti did not react in exothermic self-propagating mode. In addition to the absence of a bright flash, the nanometric grains observed in Figure 10 (<50 nm) are not consistent with a SHS reaction path. Due to the high temperatures attained, self-propagation usually comes with noticeable grain growth. Based on the enthalpy of formation of AlNi and NiTi [7], the lowest energy of Ni/Ti reactive multilayer thin films are less prone to self-propagation than the Al/Ni ones. It should be noted that the Al/Ni multilayer thin films deposited onto thin W wires presented several defects, while the Ni/Ti multilayers do not. Nevertheless, in none of the multilayer systems was a self-propagating reaction promoted upon ignition by mode I. Upon ignition by mode I, the heat released by Ni and Me reaction dissipates through the substrate and extinguishes the reaction; therefore, it does not become self-sustained.

**Figure 11:**
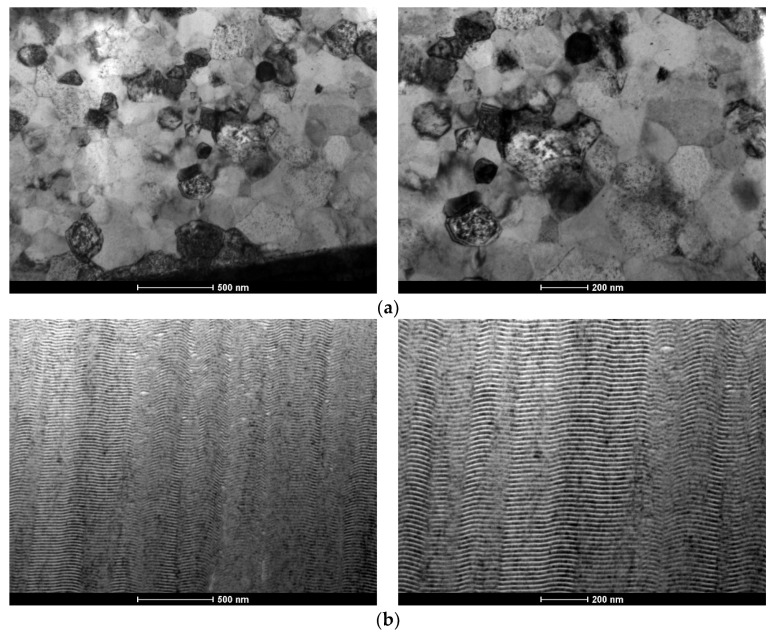
BF TEM cross-section micrographs of Al/Ni thin films after ignition by mode II. (**a**) ϕ = 0.05 mm W wire, and (**b**) ϕ = 0.20 mm W wire.

In an alternative approach, a current was applied at the uncoated ends of the W wires (ignition by mode II). In this case, self-propagation would be promoted from the interface with the substrate towards the surface of the multilayer thin films. Figure 11 shows the cross-section of Al/Ni multilayer thin films after ignition by mode II. The multilayers’ deposition parameters are the same as those used for the film shown in Figure 6. In this case, ignition of the multilayer coated W wire with ϕ = 0.05 mm was followed by a bright flash (Figure 11a). The nanolayered structure disappeared as a result of the Al and Ni reaction. In addition, the grains, whose size in the as-deposited films are limited by the individual nanolayers, grew to sub-micrometric size. Consequently, a self-propagating reaction was promoted. Using the same procedure, a similar reactive multilayer thin film deposited onto a high diameter W wire (ϕ = 0.20 mm) did not react. The Al- and Ni-rich layers are still perceptible (Figure 11b). The advantage of ignition using mode II, is that the current through the W wires can be increased to balance the increase in the diameter of the wires, or even the use of less energetic systems, such as Ni–Ti. Therefore, in future, new Ni/Me multilayer thin films will be prepared to be tested using ignition by mode II. The aim is to use these multilayer coated W wires as part of a self-healing actuator, since the heat released upon ignition should be capable of melting a repairing material, which will then flow across material failures and stop crack propagation.

**Figure 12:**
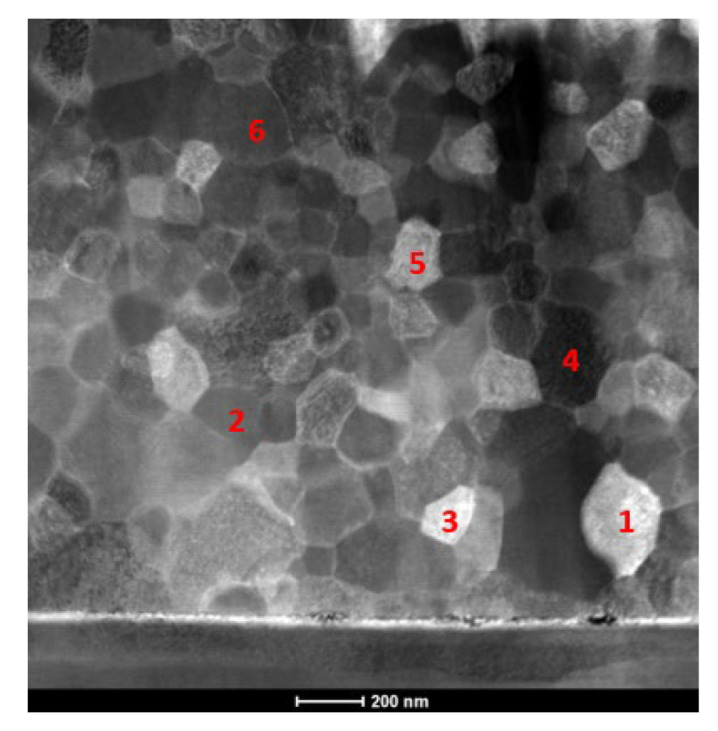
STEM micrograph of the Al/Ni thin film following ignition by mode II (ϕ = 0.05 mm W wire), indicating the points where EDS analyses were carried out.

The film presented in Figure 11a was analyzed in detail to identify the reaction product(s). Due to the grain growth, it was possible to carry out EDS analyses in individual grains (Figure 12).

**Figure 13:**
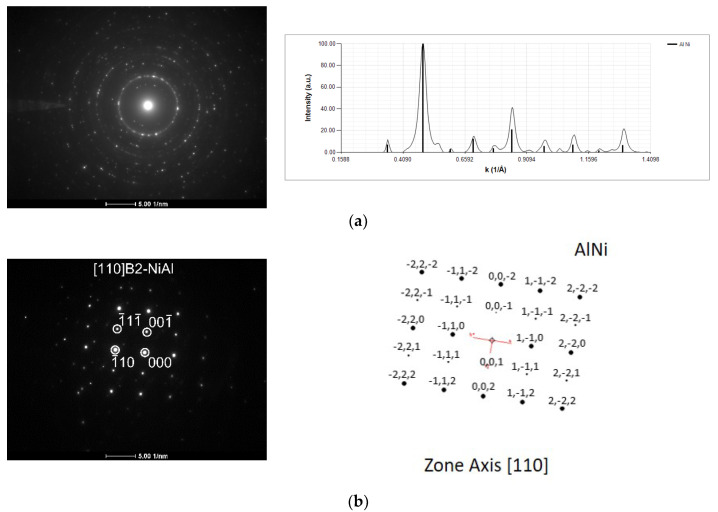
SAED patterns and respective indexation of the Al/Ni thin film after ignition by mode II (ϕ = 0.05 mm W wire). (**a**) large SAA (100 μm real diameter), and (**b**) small SAA (10 μm).

The chemical composition results (Table 1) reveal a slight Ni enrichment, even considering that vanadium exhibited a preference for the aluminum sub-lattice in AlNi [26]. Nevertheless, as planned, the presence of the AlNi equiatomic intermetallic phase was confirmed by SAED (Figure 13) using different selected area apertures (SAA). The electron diffraction pattern in Figure 13a is mainly indexed as B2-AlNi, although there are a few low intensity peaks that, considering the EDS results, might indicate the presence of a Ni-rich minor phase, such as AlNi_3_.

## 4. Conclusions

The deposition of Ni/Me (Me = Al, Ti) nanoscale reactive multilayer thin films onto small diameter wires is always a challenging task, and depending on the Ni-Me system, the problems might multiply. Based on this work, the following conclusions can be drawn:

- The nanostructured character of as-deposited Ni/Me multilayer thin films onto tungsten wires was confirmed by TEM;
- The presence of Al-rich droplets is detected by TEM in Al/Ni multilayer thin films, especially if deposited onto the smallest diameter W wires (ϕ = 0.05 mm);
- The use of a new substrate holder allowed these defects to be reduced, and more homogeneous films were obtained throughout the entire length of the wires;
- In the case of the Ni–Ti system, defect-free multilayer thin films are deposited independently of the substrate holder, wire diameter, or modulation period;
- Low energy Ni/Ti films present a fine grained microstructure, and the formation of the NiTi intermetallic phase occurred after several “shots” of ignition using mode I. However, self-propagation was not promoted;
- Using the mode II ignition, a self-propagating reaction was triggered in a W wire (ϕ = 0.05 mm) coated with an Al/Ni multilayer thin film with a modulation period close to 20–30 nm;
- During the self-propagating reaction, the desired B2-AlNi intermetallic phase formed, according to the average chemical composition of the film.

In the ignition by mode II, the electric current passes through the whole W wire, and the reaction is promoted from the interface with the substrate towards the surface of the film. Since self-propagation is accompanied by heat release, the multilayer coated W wires under study look promising for the development of actuators for self-healing applications.

## Figures and Tables

**Table 1:** EDS chemical composition (cf. Figure 12).

Point	Al [at.%]	V [at.%]	Ni [at.%]
1	42.4	3.6	54.0
2	41.0	4.4	54.6
3	36.0	4.0	60.0
4	41.3	5.0	53.7
5	40.4	4.4	55.2
6	37.4	5.0	57.6

## Data Availability

Not applicable.
